# Addressing Risks: Mental Health, Work-Related Stress, and Occupational Disease Management to Enhance Well-Being

**DOI:** 10.1155/2018/5130676

**Published:** 2018-04-15

**Authors:** Gabriele Giorgi, Jose M. Leon-Perez, Silvia Pignata, Yücel Demiral, Giulio Arcangeli

**Affiliations:** ^1^Department of Human Science, European University of Roma (UER), Rome, Italy; ^2^Department of Social Psychology, Universidad de Sevilla, Seville, Spain; ^3^Aviation Faculty School of Engineering, University of South Australia, Adelaide, SA, Australia; ^4^Department of Public Health and Department of Occupational Medicine, Dokuz Eylul University Medical Faculty, İzmir, Turkey; ^5^Department of Experimental and Clinical Medicine, University of Florence, Florence, Italy

In this special issue, Biomed Research International, section Public Health, decided to investigate the link between workers' health and work-related stress, and occupational disease management to enhance well-being.

The literature indicates that mental health and work-related stress are an increasing concern and that the management and mitigation of psychosocial risk require an interdisciplinary approach [1, 2]. In the recent international literature, mental health problems are associated with new and broader sources of work-related stress which can increase an individual's vulnerability to more serious mental health issues as well as physical and psychosomatic complaints. In addition, there is evidence of the detrimental impact of work-related stress and mental health issues on workers' health and safety, particularly with regard to cardiovascular disease, musculoskeletal disorders, and employee well-being [3, 4].

After a peer review process involving international experts, the 13 papers accepted in this special issue are empirical contributions that highlight the importance of a contextualized health approach with a focus on organizational environments. These contributions from a broad group of authors from different disciplinary sectors (organizational psychology, occupational medicine, management, nursing, medicine, epidemiology, mathematics, and mental health) represent a significant heterogeneity of countries (Asia, Australia, Eastern-Europe, Northern-Europe, South Africa, and Southern-Europe). Thus, this special issue reflects an international research perspective, which is vital for the progress of science in the field of psychosocial risk and mental health.

We highlight the importance of interdisciplinary contributions as the relationships between health sciences and work and organizations are underestimated compared to the importance given to the more general and contextualized approach of clinical health science. The concept of healthy workers in dynamic organizations that face constant issues (i.e., aging workforce, economic turbulence, and digital communication/technologies) appears to be more related to job conditions. In all of the contributions published, the concept of work contextualized health seems to emerge strongly. More than before, organizations make a critical difference when it comes to their employees' health and well-being, as they ultimately shape and contribute to their welfare.

Consequently, this special issue complements the majority of studies on mental health themes conducted in clinical, neuroscientific, and psychiatric contexts, which usually led to a person-centered analysis or research conducted in artificial laboratory settings, by offering a more ecological approach that focuses on the importance of the dimensions of “work, organizations, and occupations” in determining employees' health and well-being. Indeed, trauma and diseases related to stress and mental health that originate in the workplace may have a different pattern of development or require an organization-centered treatment approach, including field and intervention studies.

In that sense, eight out of the 13 articles published in this special issue examined (a)* antecedents and consequences of health problems and work-related stress*, while five articles provided (b)* organizational strategies and interventions to improve health and well-being*. A brief summary of each paper is presented below.

Regarding the antecedents of employees' health and well-being, S. García-Herrero and colleagues investigated occupational stress in a sample of 2,211 healthcare workers derived by the sixth European Working Condition Survey (EWCS) in their paper “The Influence of Recognition and Social Support on European Health Professionals' Occupational Stress: A Demands-Control-Social Support-Recognition Bayesian Network Model.*”* Using a Bayesian network analysis, the authors indicated that emotional demands have a greater impact on stress due to workload than family demands.

In the article, “Exploration of the Association between Nurses' Moral Distress and Secondary Traumatic Stress Syndrome: Implications for Patient Safety in Mental Health Services,” M. Christodoulou-Fella and colleagues investigated work-related moral distress (MD) and secondary traumatic stress syndrome (STSS) in association with compromised health status among health professionals. Remarkably, situations that may lead health professionals to be in moral distress seem to be principally associated with the work environment.

Also, M. Iorga and colleagues in the article “Factors Influencing Burnout Syndrome in Obstetrics and Gynecology Physicians” addressed environmental and individual antecedents on burnout syndrome among obstetric and gynecology physicians. Interestingly, both the posture adopted during medical interventions and their long working hours had important negative effects on physicians' well-being.

In the article “Leadership and Bullying in the Forestry Organization of Turkey,” M. M. Bayramoğlu and D. Toksoy examined bullying prevalence and antecedents in 1,189 forestry engineers working at 25 different Regional Directorates of Forestry in Turkey. Their results have important implications for bullying acceptability and awareness, which are crucial factors in the emergence of bullying in workplace settings.

Concerning the consequences of psychosocial risks in the workplace on both psychological and physiological health indicators at the individual level, X. Liu and colleagues in the article “The Risk Factors of High Blood Pressure among Young Adults in the Tujia-Nationality Settlement of China” concluded that hypertension has increased in China's South West province of Hubei during the last years. An interesting finding is that one of the main risk factors of such increased hypertension is associated with work: being a blue collar employee who works in rural areas.

In a similar vein, M. U. Javaid and colleagues, in the paper “Does Psychosocial Work Environment Factors Predict Stress and Mean Arterial Pressure in the Malaysian Industry Workers?” investigated the influence of psychosocial work environment factors on the health of Malaysian workers in the petrochemical industry. According to the Job Demands-Resources theory, their results revealed that psychosocial demands predicted stress and increased mean arterial pressure. These findings are particularly relevant because mental health problems and work-related stress are hot topics in the Asia-Pacific region.

In addition, stress-related issues in workplace settings are also associated with indirect indicators of organizational productivity. For example, S. Berlanda and colleagues in the article “Dissatisfaction in Child Welfare and Its Role in Predicting Self-Efficacy and Satisfaction at Work: A Mixed-Method Research” highlighted the key role of psychosocial factors such as interpersonal trust and mutual respect in predicting professional self-efficacy and job satisfaction among child welfare workers.

The article by M. Vignoli and colleagues “Workplace Phobic Anxiety as a Mental Health Phenomenon in the Job Demands-Resources Model” examined the emerging concept of workplace phobic anxiety in a nonclinical context using the Job Demands-Resources Model. As well as increasing an awareness of workplace phobic anxiety as a relevant psychosocial risk at work, the results are particularly interesting for increasing an organization's productivity because workplace phobia was found to be linked to absenteeism.

On the other hand, five articles focused on interventions in order to improve employees' health and well-being.

First, in the paper “Interventions: Employees' Perceptions of What Reduces Stress,” S. Pignata and colleagues examined qualitative information from 419 Australian employees about what measures they perceived to be effective in reducing stress among the strategies implemented at their universities. Their conclusions highlight the importance of implementing diverse multilevel strategies ranging from teaching protective coping strategies to individuals, introducing changes in job roles, and increasing recognition at the organizational level.

In an interesting longitudinal study titled “Long-Term Effectiveness of a Stress Management Intervention at Work: A 9-Year Follow-Up Study Based on a Randomized Wait-List Controlled Trial in Male Managers,” J. Li and colleagues offer evidence on the long-term effectiveness of an 18-hour psychotherapeutic stress management intervention (SMI) in the workplace over a 9-year period. This intervention, rooted in the solid theoretical framework of the Effort Reward Imbalance (ERI) model of work stress, seems to be a promising and inspiring tool for developing work contextualized mental health programs.

The paper “Exposure to Workplace Bullying: The Role of Coping Strategies in Dealing with Work Stressors” authored by W. Van den Brande and colleagues pointed out the interaction between work stressors (i.e., workload, job insecurity, role conflict, and role ambiguity) and employees' coping strategies (i.e., problem- and emotion-focused) in predicting exposure to workplace bullying. Their results identified a potential prevention area at both individual and organizational levels.

Y.-T. Ke and colleagues, in the paper “Posttraumatic Psychiatric Disorders and Resilience in Healthcare Providers following a Disastrous Earthquake: An Interventional Study in Taiwan,” argued that posttraumatic psychiatric disorders were common in healthcare providers following a medical response to an earthquake. However, the introduction of an early intervention that added muscle and mental relaxation tools to the more classic psychotherapeutic measures increased employees' resilience and reduced posttraumatic stress symptoms one month after the natural disaster in Taiwan.

Finally, the results from the study by Z. Gong and colleagues “How to Apply Feedback to Improve Subjective Wellbeing of Government Servants Engaged in Environmental Protection in China?” showed that supervisor feedback, through the fulfillment of basic psychological needs satisfaction, played a key role in increasing the subjective well-being of government servants engaged in environmental protection roles. The implications are particularly relevant in high power distance cultures.

In conclusion, we hope that this special issue will inspire academics and practitioners to consider addressing the specific organizational aspects that may be created or exacerbated by work rather than overestimating individual behavior, personality, and psychiatric syndromes. Well-being lies not only in the person, but also in the organization in which s/he works.
